# Induction of Endoplasmic Reticulum-Derived Replication-Competent Membrane Structures by West Nile Virus Non-Structural Protein 4B

**DOI:** 10.1371/journal.pone.0084040

**Published:** 2014-01-20

**Authors:** Pakieli H. Kaufusi, James F. Kelley, Richard Yanagihara, Vivek R. Nerurkar

**Affiliations:** 1 Department of Tropical Medicine, Medical Microbiology and Pharmacology, John A. Burns School of Medicine, University of Hawaii at Manoa, Honolulu, Hawaii, United States of America; 2 Department of Pediatrics, John A. Burns School of Medicine, University of Hawaii at Manoa, Honolulu, Hawaii, United States of America; 3 Pacific Center for Emerging Infectious Diseases Research, John A. Burns School of Medicine, University of Hawaii at Manoa, Honolulu, Hawaii, United States of America; The Pirbright Institute, United Kingdom

## Abstract

Replication of flaviviruses (family Flaviviridae) occurs in specialized virus-induced membrane structures (IMS). The cellular composition of these IMS varies for different flaviviruses implying different organelle origins for IMS biogenesis. The role of flavivirus non-structural (NS) proteins for the alteration of IMS remains controversial. In this report, we demonstrate that West Nile virus strain New York 99 (WNV_NY99_) remodels the endoplasmic reticulum (ER) membrane to generate specialized IMS. Within these structures, we observed an element of the cis-Golgi, viral double-stranded RNA, and viral-envelope, NS1, NS4A and NS4B proteins using confocal immunofluorescence microscopy. Biochemical analysis and microscopy revealed that NS4B lacking the 2K-signal peptide associates with the ER membrane where it initiates IMS formation in WNV-infected cells. Co-transfection studies indicated that NS4A and NS4B always remain co-localized in the IMS and are associated with the same membrane fractions, suggesting that these proteins function cooperatively in virus replication and may be an ideal target for antiviral drug discovery.

## Introduction

The West Nile virus (WNV) genome consists of a single-stranded, positive-sense RNA of approximately 11 kb that encodes a single polyprotein precursor, which is processed by cellular and viral-encoded proteases into three structural proteins and seven non-structural (NS) proteins. The roles of NS proteins in the WNV life cycle are known [1], except for NS4B, the largest of the small hydrophobic NS proteins of flaviviruses, which consists of three endoplasmic reticulum (ER) membrane-spanning segments. NS4B of WNV strain Kunjin (WNV_KUN_) can translocate to the nucleus of infected cells, yet its function in the nucleus is unknown [2], [3]. The NS4B of dengue virus type 2 (DENV-2) also spans the ER and is part of the membrane-bound viral replication complex [4]. Despite its ability to inhibit the host interferon (IFN) antiviral response [5], [6] and induce monocyte-derived inflammatory cytokines [7], NS4B may play a more direct role in viral RNA replication and pathogenesis as suggested by numerous NS4B mutational studies [8], [9], [10], [11], [12].

Replication and assembly of the plus-strand flaviviruses rely on the remodeling of intracellular membranes into several characteristic structures, including convoluted membranes (CM), paracrystalline (PC) arrays, and vesicle packets (VP) or smooth membrane structures [13], collectively referred to in this study as induced membrane structures (IMS). Previous studies have established spatial and functional relationships between several viral NS proteins and IMS [14], [15], [16], [17]. However, the precise role of NS proteins during IMS formation in flavivirus-infected cells remains poorly understood. Moreover, it is unclear which cellular organelle membranes are exploited by viral proteins during IMS biogenesis. For DENV-2 and WNV_KUN_, it has been speculated that the proteins within the polyprotein NS4A-2K-NS4B are responsible for remodeling infected cell membranes [4], and that regulated processing of NS4A-2K-NS4B to release NS4A and NS4B proteins is critical for IMS formation [2], [4]. NS4A is a small hydrophobic protein [18] that contains multiple membrane-spanning regions [19]. NS4A of WNV_KUN_ is proposed to be responsible for IMS formation [2], [19], which is derived from the *trans*-Golgi network [20]. However, DENV-2 NS4A may induce IMS derived from the ER [4]. Therefore, NS4A may initiate IMS formation from different cellular organelles or sub-organelles, depending on the flavivirus species.

Previous studies demonstrate that the flavivirus NS4A-2K-4B polyprotein, which has a 2-kDa signal peptide of 17–23 amino acids at the COOH-terminal end of NS4A protein [21], undergoes cleavage and processing in a highly regulated and sequential manner [4], [19]. Initially, the viral NS2B-3pro cleaves the NS4A-2K-4B polyprotein precursor to release NS4A from 2K-NS4B. It is not until after the initial cleavage that a host protease cleaves away 2K and releases mature NS4B protein [4], [19]. During this sequential processing, NS4B integrates into the ER membrane. Interestingly, the 2K-signal peptide is not required for DENV-2 NS4B integration into the ER membrane [4]. However, the 2K is important during IMS formation as part of WNV_KUN_ NS4A [2], [21] but not DENV-2 NS4A [19]. Moreover, it is thought that the 2K plays a direct role in flavivirus RNA synthesis, as suggested in the WNV 2K mutational study demonstrating resistance to lycorine, a flavivirus inhibitor [22]. Collectively, these studies provide conflicting evidence surrounding the role of the 2K-signal peptide on flavivirus NS4B localization and function, and warrant further investigation. The focus of this study was to investigate the origin of the cellular membranes utilized by WNV strain NY99 (WNV_NY99_) and the role of NS4B protein in IMS biogenesis, using confocal immunofluorescence microscopy (IFM) and biochemical assays.

## Results

### WNV_NY99_ IMS are derived from the ER and not from the early endosome or trans-Golgi

**Figure 1:**
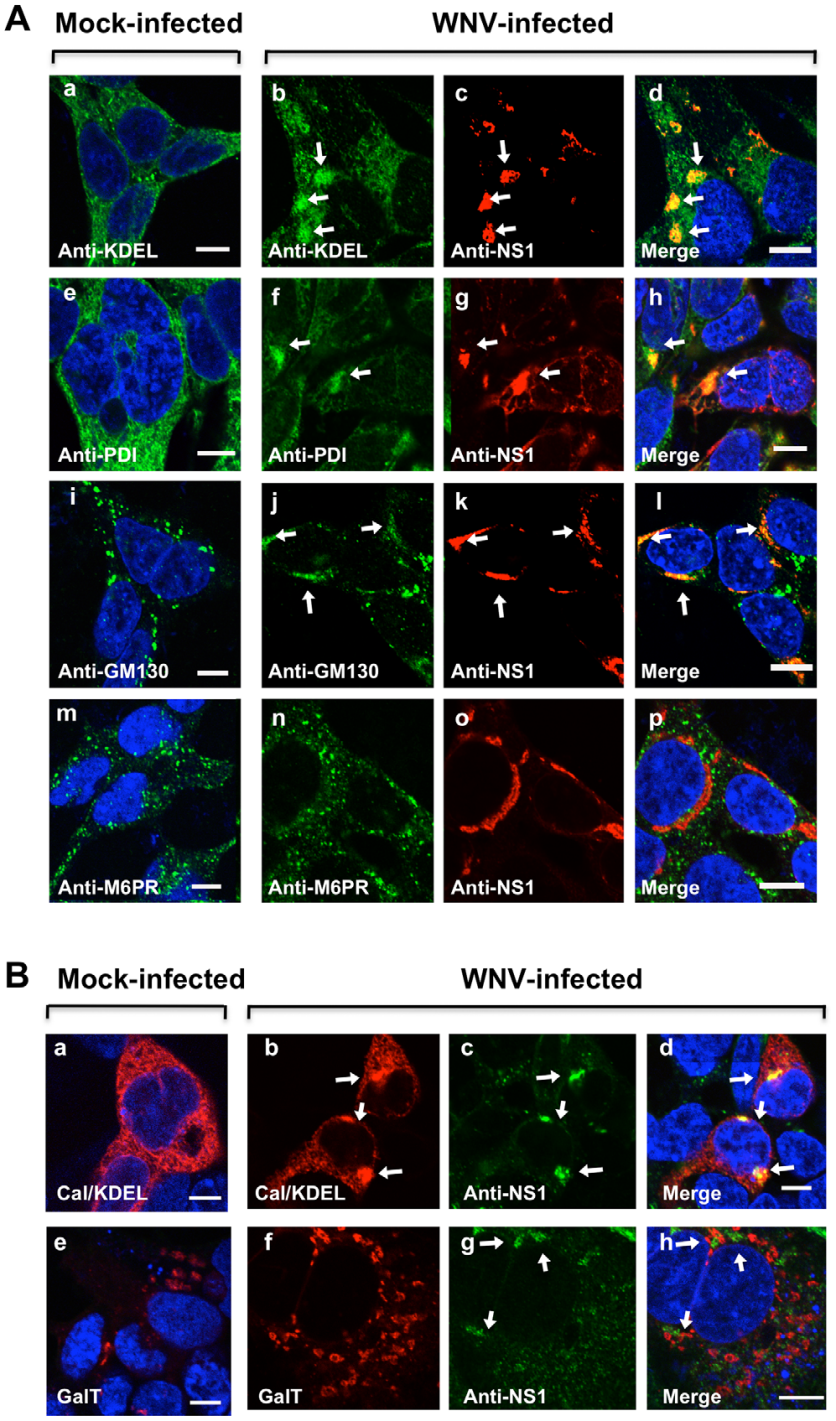
WNV NS1 antigen and IMS are localized to the endoplasmic reticulum (ER) and *cis*-Golgi organelles. (A) HEK293 cells were infected with WNV and after 24 hr, cells were fixed and immunostained with mouse anti-KDEL (a and b) and rabbit anti-PDI (e and f) polyclonal antibodies to detect the ER, mouse anti-GM130 to detect the *cis*-Golgi (i and j) and mouse anti-M6PR to detect early endosomes (m and n). The cells were washed and co-immunostained with WNV mouse anti-NS1 antibody (red; c, d, g, h, k, l, o and p). (B) HEK293 cells were transfected for 12 hr with pDsRed2-ER containing the ER targeting sequence of calreticulin and the ER retention sequence, KDEL (a, b, and d) or pDsRed-Monomer-Golgi encoding the N-terminal 81 amino acids of human beta 1,4-GalT, (e, f, and h). After 2 hr, cells were infected for 24 hr with WNV (b–d and f–h), fixed and co-immunostained with WNV mouse anti-NS1 antibody (green; c, d, g, and h). The nuclei were counterstained by 4,6-diamidino-2-phenylindole (DAPI). Arrows indicate the IMS. Confocal fluorescence images were of optical slice thickness ∼1 µm. Scale bar, 10 µm.

Previous studies suggest that the intracellular membranes for IMS biogenesis appear to differ across flaviviruses [4], [19], [20] and the discrete virus-IMS are readily identifiable by confocal IFM [4], [13], [20]. To define the cellular origins of membranes utilized by WNV_NY99_ for IMS biogenesis, we conducted a dual-labeling assay using fixed HEK293 cells at 24 hr after infection and antibodies specific for WNV_NY99_ NS1, a component of the flavivirus-IMS [17], [23], and subcellular marker proteins (Fig. 1). Utilizing high-resolution confocal IFM, we observed ER proteins containing carboxy-terminal KDEL motif responsible for ER retention [24], [25], and protein disulfide isomerase (PDI), a soluble protein resident of the ER [26], which localized to the IMS in infected cells, as defined by the presence of NS1 protein (Fig. 1A, b–d, f–h and, j–l). Interestingly, GM130, a peripheral membrane protein localized primarily at the *cis*-face of the Golgi apparatus [27], also localized to the IMS (Fig. 1A, j–l); whereas mock-infected cells showed no evidence of IMS formation (Fig. 1A, a, e, i). The co-localization of GM130 protein to the IMS suggested that a component of the *cis*-Golgi compartment was recruited to the IMS. Conversely, there was no apparent co-localization of the mannose-6-phosphate receptor (M6PR), a marker for early endosome [28], with NS1 (Fig. 1A, n–p) indicating that the membrane for WNV_NY99_ IMS formation was not derived from this organelle. Additionally, we demonstrated the absence of the *trans-*Golgi marker by expressing a fluorescent protein-tagged human 1,4-galactosyltransferase (GalT) [29] (Fig. 1B, f–h), which functionally mimics the native GalT, and further confirmed the presence of ER-derived IMS by using the calreticulin/KDEL-dsRed, a fluorescent protein-tagged version of the ER with the retention sequence KDEL [29] (Fig. 1B, b–d).

### NS4B protein is localized to the IMS in WNV-infected cells

**Figure 2:**
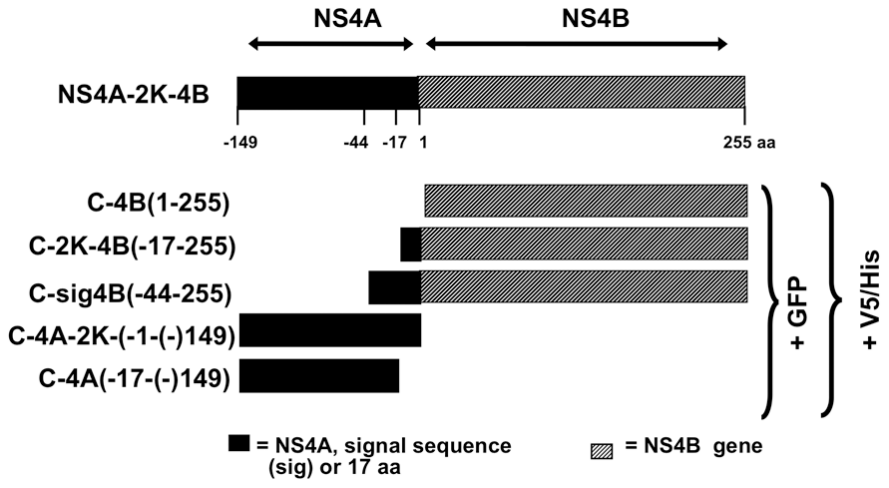
Schematic representation of plasmid constructs used in this study. The full-length NS4A-2K-NS4B (C-4AB) is depicted with amino acid (aa) numbers, 1 representing the first aa at the N-terminus of NS4B. Also depicted are the plasmids NS4B (C-4B), NS4B retaining 17 N-terminal aa (C-2K-4B), NS4B retaining 44 N-terminal aa (C-sig4B), NS4A retaining the 17-aa to the C-terminal segment (C-4A-2K), and NS4A without the 2K-signal peptide (C-4A). NS4A and the 44- or 17-aa preceding C-4B are indicated by a black box. Plasmid fusion detection peptides, either GFP or V5-His epitope, are noted to the right.

To unravel the role of WNV_NY99_ NS4B in IMS biogenesis, we constructed several expression cassettes encoding various lengths of NS4A plus NS4B protein fused to GFP or V5/His epitopes (Fig. 2) as described previously [7], [30]. One construct contained the NS4B protein, designated C-4B, and two additional cassettes carried 17 residues, designated C-2K-4B, or 44 residues, designated C-sig4B, of NS4A encoding for a 2K-signal peptide important for ER localization of NS4B [21]. To ensure validity and reliability of our expression system where the plasmid is expressed alone or introduced into infected cells [28], we constructed two expression cassettes encoding the NS4A protein retaining the signal peptide, designated as C-4A-2K, or lacking the signal peptide, designated as C-4A. The NS4A cassettes were verified to localize to the IMS in WNV-infected cells (data not shown), consistent with a previous report [19], and thereby validates the gene expression system of transfection together with WNV infection adopted in this study [28].

**Figure 3:**
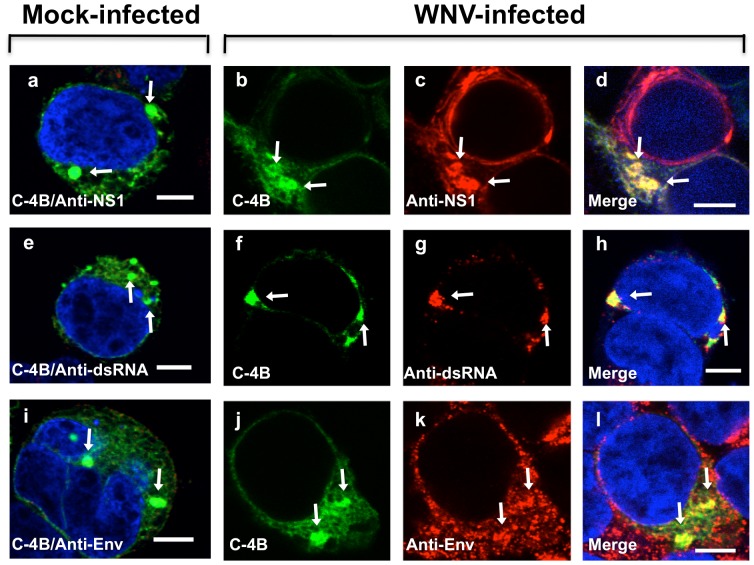
Localization of WNV NS4B to IMS in infected cells. HEK293 cells were infected with WNV and after 2-GFP plasmid (C-4B; b–d, f–h, and j–l). Slice confocal IF images of (a, e, and i) mock-infected but transfected, and (b–d, f–h, and j–l) WNV-infected and transfected fixed HEK293 cells at 24 hr immunostained with WNV mouse anti-NS1 antibody (red; c–d), mouse anti-dsRNA antibody (red; g–h), or WNV mouse anti-envelope antibody (red; k–l). The mock-infected cells were transfected with the NS4B-GFP plasmid and immunostained with the indicated antibodies (a, e, and i). Nuclear DNA was labeled with DAPI. The arrowheads indicate IMS. Confocal IF images were of optical slice thickness ∼1 µm. Scale bar, 10 µm.

To determine whether WNV_NY99_ NS4B localizes to the IMS, characterized in Fig. 1 and defined by components of the flavivirus replication complexes including the replicating double-stranded RNA (dsRNA) [4], [19], NS1 protein [16], [17], and envelope protein [19], we conducted a co-localization assay on fixed cells by confocal IFM at 24 hr after infection (Fig. 3). HEK293 cells were infected with WNV_NY99_ at MOI 1 for 2 hr, then transfected with the NS4B-GFP (C-4B) plasmid (Fig. 3, b–d, f–h and j–l). The control cells were transfected but not infected (Fig. 3, a, e and i). The specificity of the antibodies was determined by staining the uninfected cells with mouse monoclonal anti-NS1 (Fig. 3, a; red), anti-dsRNA (Fig. 3, e; red), or anti-envelope (Env) (Fig. 3, i; red). In these cells, no red fluorescence was observed but NS4B foci, referred to as NS4B-IMS, were noted throughout the cytoplasm as large foci in the perinuclear region. These NS4B-IMS appeared very similar to virus-IMS observed in infected cells (Fig. 3). Extensive co-localization of NS4B-GFP and viral NS1 or viral dsRNA (Fig. 3, d and h) to WNV_NY99_ ER-derived IMS (Fig. 1A, b–d and f–h) was observed. The NS4B-GFP also co-localized with WNV_NY99_ envelope protein (Fig. 3, i). WNV_NY99_ NS4B was not detected in the nucleus (Fig. 3, d, h, i) and the same result was found in human neuroblastoma cells (SK-N-SH) (data not shown), suggesting that the non-nuclear localization of WNV_NY99_ was not cell-type specific.

### NS4B with and without 2K signal peptide induces membrane structures morphologically similar to virus-IMS

**Figure 4:**
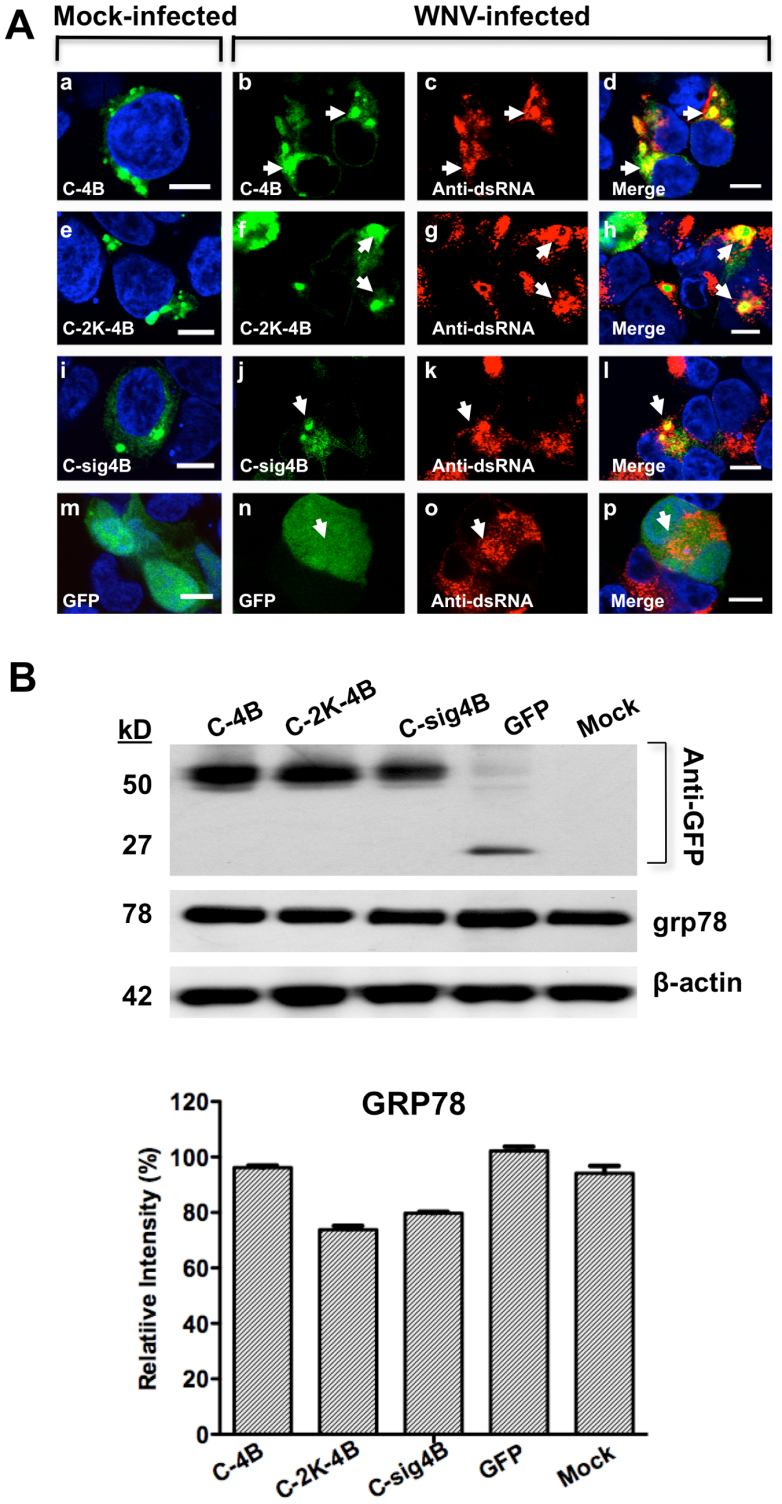
IMS localization and expression of WNV NS4B-GFP with or without the 2K-signal peptide in infected cells. (A) HEK293 cells were infected (b–d, f–h, j–l, and n–p) or mock-infected (a, e, i, and m) with WNV and after 2 hr transfected with C-4B (b–d), C-2K-4B (f–h), C-sig4B (j–l) or GFP (n–p). Cells were fixed at 24 hr after infection and immunostained with mouse anti-dsRNA antibody (red; c–d, g–h, k–l and o–p). The mock-infected cells were transfected and immunostained with mouse anti-dsRNA antibody (a, e, i, and m). Nuclear DNA was labeled with DAPI. The arrowheads indicate IMS. Confocal IF images were of optical slice thickness ∼1 µm. Scale bar, 10 µm. (B) Western blot analysis of WNV NS4B plasmids and stress response in transfected HEK293 cells. Fifty µg of cell lysate was loaded and electrophoresed on SDS-PAGE followed by immunoblotting with rabbit anti-GFP polyclonal antibody, an anti-GRP78 monoclonal antibody or anti-β-actin monoclonal antibody followed by a peroxidase-conjugated secondary antibody. ImageJ analysis of the western blot was conducted to determine GRP78 protein levels relative to β-actin.

To investigate the role of the 2K-signal peptide preceding the WNV_NY99_ NS4B, we conducted a co-localization assay on fixed cells by confocal IFM at 24 hr after infection (Fig. 4A). HEK293 cells were infected with WNV_NY99_ followed by transfection with the NS4B-GFP lacking (C-4B) (Fig. 4A, b–d) or retaining the 2K-signal peptide (C-2K-4B or C-sig4B; Fig. 4A, f–h or Fig. 4A, j–l, respectively), as described in the previous section. The control cells were infected, followed by transfection with GFP plasmid (Fig. 4A, n–p). In the control cells, GFP did not co-localize with the viral dsRNA to the IMS indicating the specificity of the components of the IMS in the infected cells. As we observed in Fig. 3, NS4B localized to the dsRNA IMS (Fig. 4A, b–d). A comparable localization was also observed with the NS4B retaining the 2K (Fig. 4A, f–h and j–l) suggesting that the 2K-signal peptide has no clear role on the localization of the NS4B protein to the virus-IMS. Also, large perinuclear IMS resembling dsRNA IMS observed in infected cells were observed in mock-infected cells expressing NS4B lacking (Fig. 4A, a) or retaining the 2K-signal peptide (Fig. 4A, e and i) suggesting that the 2K-signal peptide plays a minimal role in the induction of virus-IMS. Expression of various NS4B (C-4B, C-2K-4B, or C-sig4B) or GFP proteins was confirmed by western blotting (Fig. 4B, top panel). Control GFP and NS4B-GFP bands migrated to their expected molecular weights, 27- and 52-kDa, respectively. The 2K signal peptide is expected to pull the N-terminal end of NS4B into the lumen to be cleaved by the host signalase [4], [7]. Indeed, NS4B retaining the 2K signal peptide (C-2K-4B) produced a 52-kDa band, indicating that a host signalase cleaved 2K from NS4B. Interestingly, NS4B encompassing the 2K-signal peptide within the 44 aa to the C-terminal end of NS4A (C-sig4B) produced the same size band as NS4B, indicating that this truncated polyprotein was appropriately processed to release the NS4B.

To rule out the possibility that NS4B-IMS is a side effect of ER stress-induced apoptosis, we measured the expression of the glucose response protein 78 (grp78), a molecular chaperone required for ER integrity and stress-induced autophagy [33] (Fig. 4B, top panel). We compared the intensity of grp78 bands to the intensity of β-actin control bands, assigned an arbitrary standard value of 100%, and graphed relative percentages (Fig. 4B, bottom panel). There was no significant increase in the production of grp78 in cells expressing NS4B lacking or retaining the 2K-signal peptide than was observed in mock-transfected or GFP-expressing cells (Fig. 4A, a, e and i).

Because flavivirus NS4B is associated with the intracellular membrane [4], it is possible that the NS4B-IMS in the transfected cells (Fig. 4A) is a general feature of overexpressed membrane proteins. To test this possibility, we conducted time-course experiments on HEK293 cells transfected with two expression cassettes, one encoding a selenoprotein K (SelK), an ER membrane protein important for efficient Ca^2+^ flux during the activation of immune cells, fused to GFP [34], and the other a calreticulin/KDEL-dsRed (Cal/KDEL), a fluorescent membrane protein-tagged version of the ER with the retention sequence KDEL [29]. Utilizing high-resolution IFM, we observed no evidence of IMS formation in SelK (Fig. S1A, top panel) or Cal/KDEL (Fig. S1B, top panel) expressing cells. It is important to note that the IMS are not present even with relative increase of SelK (Fig. S1A, bottom panels) or Cal/KDEL (Fig. S1B, bottom panels) expression suggesting that the IMS is not a general feature of overexpressed membrane protein.

### The NS4B-induced membrane structure is found independent of NS4B concentration

**Figure 5:**
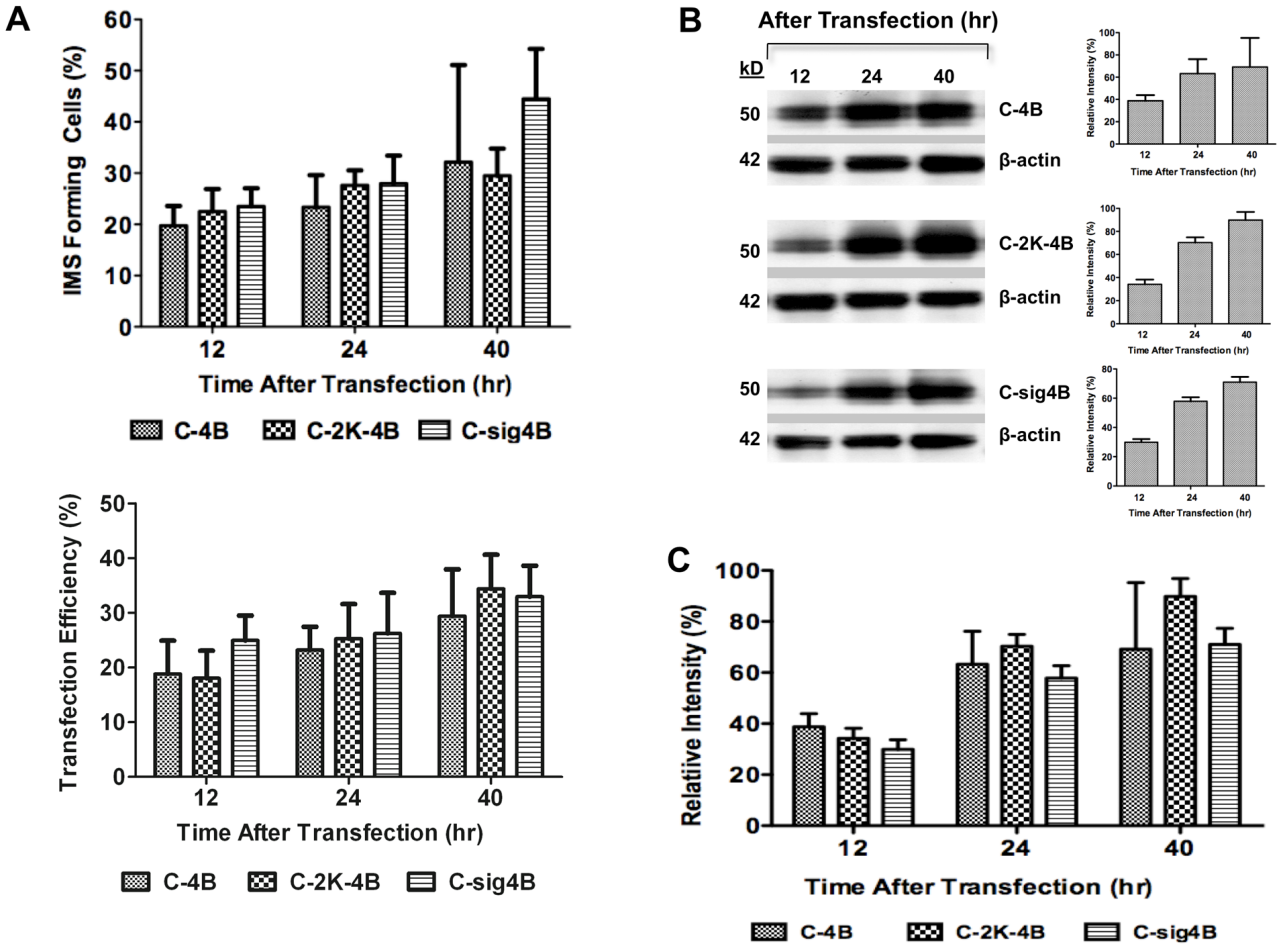
Quantitation of IMS in HEK293 cells expressing NS4B with and without the 2K-signal peptide. (A) Average number of IMS forming cells at 12, 24 and 40 hr after transfection. Each data point represents the percent of IMS forming cells in 50–100 GFP positive cells in 10 fields from two independent transfections. The average percent transfection efficiency for each data point is shown for 12, 24 and 40 hr after transfection. (B) HEK293 cells were transfected with a NS4B-GFP plasmid with and without the 2K signal peptide and harvested at 12, 24 and 40 hr after transfection. Fifty µg of harvested cell lysates were electrophoresed on SDS-PAGE followed by immunoblotting with rabbit anti-GFP antibody followed by a peroxidase-conjugated secondary antibody. The western blot images were analyzed using ImageJ to determine the relative intensity of the GFP expression level relative to β-actin, depicted as a percent relative intensity. (C) The average relative intensity of NS4B with the 2K signal peptide (C-2K-4B and C-sig4B) and without 2K (C-4B) at 12, 24 and 40 hr after transfection were determined using ImageJ.

It has been proposed that the induction of HCV NS4B-induced structures is dependent on achieving a critical intracellular concentration of NS4B [35]. We transfected HEK293 cells with equal amounts of WNV_NY99_ NS4B plasmids and evaluated NS4B-IMS formation as a result of increased intracellular concentrations of NS4B with or without the 2K-signal peptide at predetermined time points. IMS were not evident in the control GFP-expressed cells. We observed an increased trend in NS4B-IMS forming cells from 12 to 40 hr after transfection for all three NS4B variants but the increase was not significant (Fig. 5A, top panel); each plasmid expressed with similar efficiency (Fig. 5A, bottom panel). The induction appeared to be independent of the intracellular concentration of NS4B protein (Fig. 5B and C). Additionally, NS4B retaining the 2K-signal peptide did not influence the number of NS4B-IMS despite the small increase at 24 to 40 hr in cells expressing the C-sig4B further supporting our previous finding that the 2K-signal peptide plays minimal role in IMS formation (Fig. 5A).

### NS4B protein is integrated into the ER membrane

**Figure 6:**
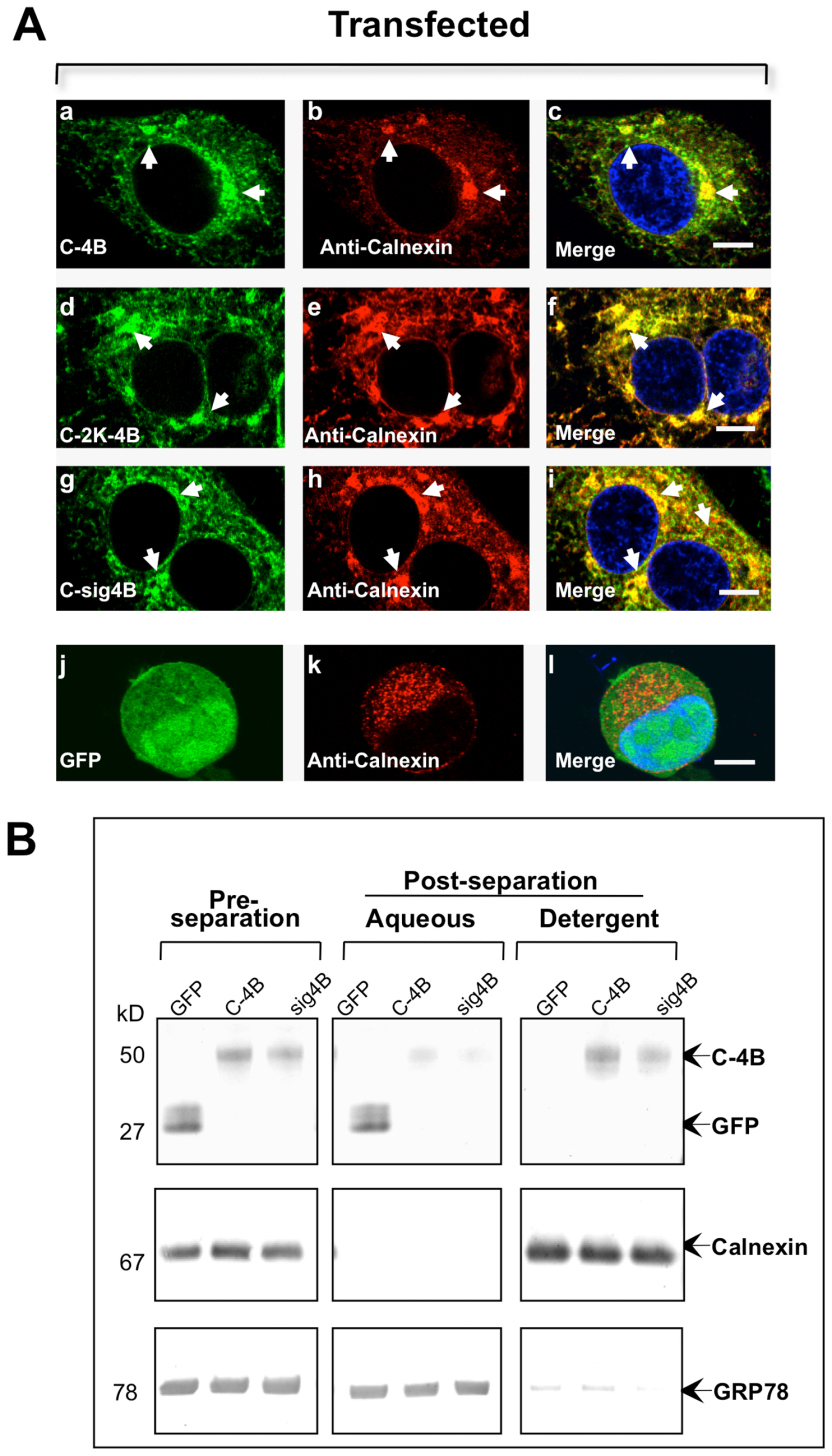
Co-localization of WNV NS4B lacking or retaining the 2K-signal peptide with the ER marker, calnexin, to NS4B-IMS. (A) Transfected HEK293 cells were fixed after 24 hr and processed for IF analysis. Left panels (a, d, g, j) depict different NS4B-GFP fusion constructs, and panels in the middle column (b, e, h, k) depict cells stained with calnexin. Merged images are depicted in the right panels (c, f, i, l). The arrowheads indicate IMS co-localized with calnexin; GFP alone does not co-localize with calnexin (l). (B) NS4B is associated with the cellular membrane. Triton X-114 phase separation of the total cell lysates from HEK293 cells transfected for 24 hr with GFP, NS4B (C-4B) or NS4B-retaining 2K (C-sig4B) plasmid. Total cell lysates were subjected to SDS-PAGE, immunostained against GFP (top panel), anti-calnexin (middle panel), anti-GRP78 (bottom panel) and visualized with AP-conjugated secondary antibodies. Twenty-five µL of total protein was loaded into each lane.

The WNV-IMS is clearly derived from the ER membrane in infected cells (Fig. 1), and the NS4B-IMS in transfected cells (Fig. 4A) resembles WNV-IMS suggesting that NS4B-IMS derives from the ER. We next transfected HEK293 cells with the NS4B gene plasmids used in Fig. 4 and conducted a co-labeling assay with the ER membrane marker calnexin to establish the origin of membranes for NS4B-IMS and evaluated the role the 2K-signal peptide in NS4B localization. Consistent with the WNV ER-derived IMS, we demonstrated that calnexin co-localized with NS4B-IMS (Fig. 6A, c) but not with GFP in the control cells (Fig. 6A, l). Additionally, the calnexin protein was localized to the NS4B-IMS produced by all three independently expressed NS4B proteins, lacking (C-4B) or retaining the 2K-signal peptide (C-2K-4B or C-sig4B), suggesting that NS4B localizes primarily to the ER.

While DENV NS4B associates with the ER membrane [4] and WNV_KUNV_ NS4B is found in the nucleus [3], the association of WNV_NY99_ NS4B with the membrane remains unclear. The computer-based SOSUI analysis suggested that WNV_NY99_ NS4B associates with the membrane (Fig. S2). To confirm this, we conducted biochemical analysis using phase separation with Triton X-114 from HEK293 cells transfected with GFP, C-4B or C-sig4B. Even though we have not seen phenotypic difference between C-2K-4B and C-sig4B plasmids on any of our assays, we used the C-sig4B plasmid instead of the C-2K-4B to ensure that all amino acids of the 2K-signal peptide were retained. As expected, we detected the control GFP protein in the aqueous phase, while NS4B with and without 2K was detected in the detergent phase (Fig. 6B) indicating that both viral proteins were integrated in the membrane. The control ER membrane protein, calnexin, was detected in the detergent phase and the ER soluble protein, grp78, was predominantly detected in the aqueous phase (Fig. 6B). Collectively, these data suggest that WNV_NY99_ NS4B-IMS is derived from the ER and the NS4B protein is a membrane protein.

### NS4A is a component of the WNV-IMS and NS4B-IMS

**Figure 7:**
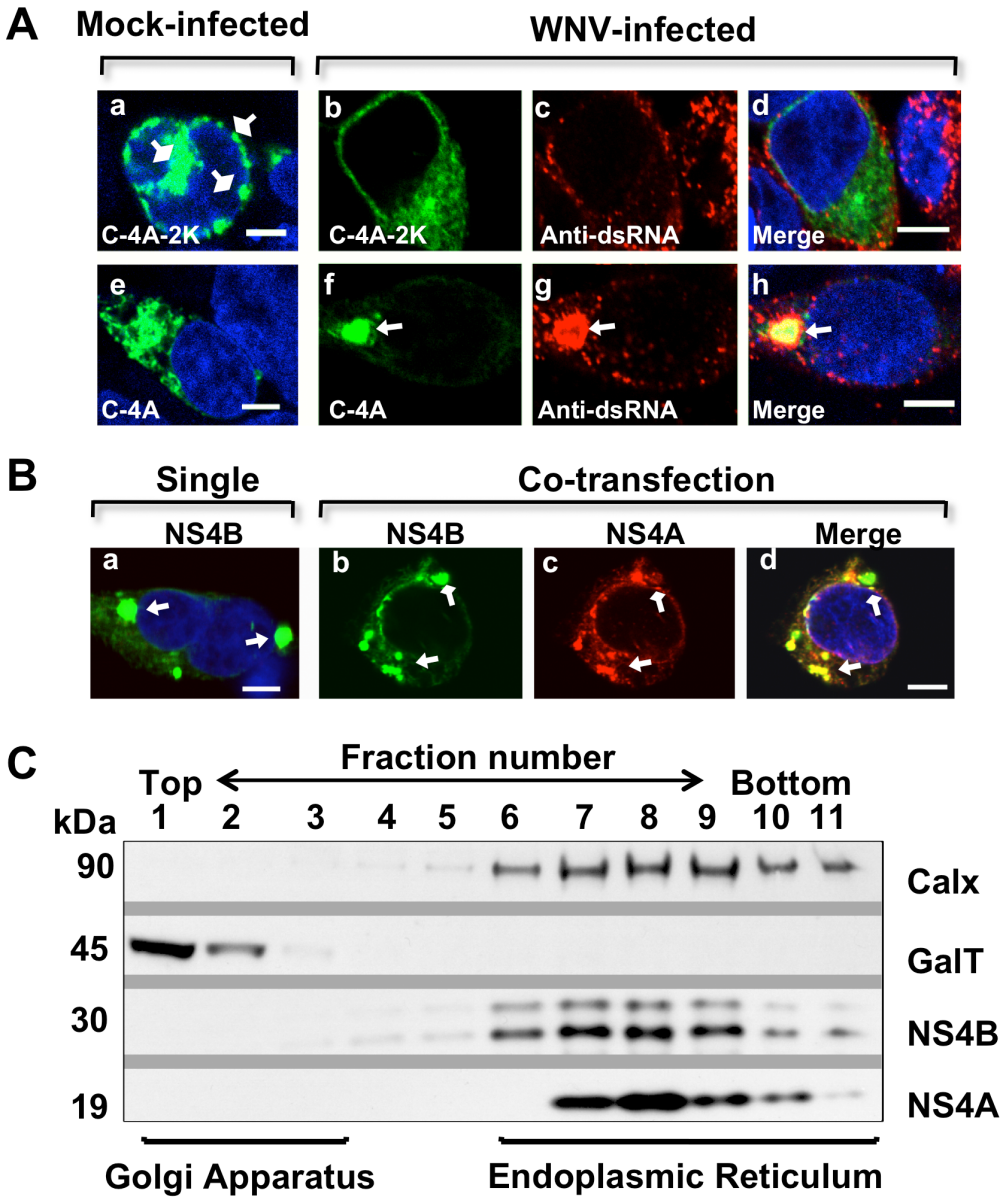
Association of WNV NS4A and NS4B proteins in IMS formation. (A) HEK293 cells were infected with WNV and after 2 hr transfected with NS4A-2K-GFP (C-4A-2K, b–d) or NS4A-GFP plasmid (C-4A, f–h). Cells were fixed at 24 hr after infection and immunostained with mouse anti-dsRNA antibody (red; c–d and g–h). The mock-infected cells were transfected with (a) C-4A-2K or (e) C-4A and were immunostained with mouse anti-dsRNA antibody (a and e). Confocal IF images were of optical slice thickness ∼1 µm. The arrowheads indicate IMS whereas the square arrowhead indicates structures (potential artifacts) induced by NS4A retaining the 2K (a). Scale bar, 5 µm. (B) IF of NS4A and NS4B localization in transfected cells. HEK293 cells were co-transfected with NS4A-V5 (C-4A) and NS4B-GFP plasmids (C-4B), (b–d) or with NS4B-GFP only (a). Cells were fixed at 24 hr after transfection and immunostained with mouse anti-V5 antibody (red, b and c). Merge image (d). Confocal immunofluorescence images were of optical slice thickness ∼1 µm. The arrowheads indicate IMS. Scale bar, 5 µm. (C) Subcellular fractionation of HEK293 cells expressing NS4A and NS4B in transfected cells. HEK293 cells were co-transfected with NS4A-V5/His and NS4B-V5/His plasmids. Cell fractions were separated using sucrose gradient and subjected to SDS-PAGE before immunoblotting with a mouse monoclonal anti-V5 antibody and HRP-conjugated secondary antibody. The ER and Golgi apparatus were detected using antibodies against the markers calnexin (Calx) and 1,4-galactosyltransferase (GalT), respectively.

While we have demonstrated that NS4B-IMS resembles WNV-IMS, previous studies demonstrate that the NS4A protein is responsible for the formation of flavivirus-IMS [2], [19] and its retainment of 2K is important for this induction [2]. Therefore, we assessed the localization pattern of WNV_NY99_ NS4A-GFP fusion either retaining (Fig. 2; C-4A-2K) or lacking the 2K (Fig. 2; C-4A) to the virus-IMS during infection. In contrast to WNV_KUN_ [2], WNV_NY99_ NS4A retaining 2K did not localize to the IMS at 24 hr, as defined by presence of viral dsRNA, but rather formed diffused fluorescent patterns in the cytoplasm (Fig. 7A, b–d). However, similar to DENV [19], NS4A lacking 2K resulted in NS4A redistribution to the WNV-IMS (Fig. 7A, f–h) suggesting that WNV_NY99_ NS4A is a component of the virus-IMS during infection but only when the 2K is lacking. It is important to note that the expression of NS4A retaining 2K induced multiple membrane structures (Fig. 7A, a) and these structures were lacking when the plasmid (C-4A-2K) was introduced into infected cells (Fig. 7A, b–d) suggesting that the multiple membrane structures are not part of the WNV-IMS.

As the multiple membrane structures induced by NS4A retaining the 2K are distinctly different from the WNV-IMS, we explored the localization of NS4A lacking the 2K to the NS4B-IMS. HEK293 cells were co-transfected with NS4A and NS4B plasmids, C-4A and C-4B, respectively, and the NS4B-IMS was assessed using confocal microscopy. We observed that NS4A was clearly localized to some but not all of the NS4B-IMS (Fig. 7B, b–d). Identical IMS were also observed in cells transfected with NS4B plasmid alone (Fig. 7B, a) implying that NS4B may initiate the IMS (Fig. 7B, b–d) and then recruit NS4A to these structures. The exact role of each protein and sequential recruitment during IMS formation warrants further investigation.

We further examined the association of NS4A and NS4B with the intracellular membranes by fractionation of cell lysates prepared from HEK293 co-transfected with NS4A and NS4B plasmids, using sucrose density gradient centrifugation [36], [37]. Western blot analysis of solubilized fractions demonstrated that the majority of NS4A and NS4B proteins were in fractions 7–9, together with the ER as indicated by its marker, calnexin, but not in fractions 1 and 2 with the Golgi apparatus as indicated by its marker, GalT (Fig. 7C). These results indicate that, in HEK293 cells, both NS4A and NS4B are associated with the ER membranes further supporting our confocal IF data (Fig. 6A).

## Discussion

Upon infection, flaviviruses cause dramatic alterations of intracellular membranes leading to the formation of IMS, which provide a platform for viral RNA synthesis and virus assembly [38]. The origin of the membranes and the viral protein(s) responsible for IMS biogenesis appear to be quite variable among members of the genus *Flavivirus*. The WNV_KUN_ protease NS2B-3pro, the polyprotein NS4A-2K-4B, and NS4A initiate IMS formation; however, WNV_KUN_ NS4B does not have IMS induction capacity [2], [19], [39]. In this report, we demonstrate that WNV_NY99_ NS4B alters the ER membrane to generate IMS, suggesting that NS4B may play a more direct role during IMS biogenesis compared to that reported for other flaviviruses [2], [19], [39]. Using HEK293 cell-based infection/transfection strategy, confocal IFM and biochemical assays, we demonstrate that WNV_NY99_ NS4B is a component of the ER-derived IMS and may be the key viral protein for IMS biogenesis.

### Alteration of the ER membrane in the WNV_NY99_-infected HEK293 cells

The IMS induced by virus infection originate from membranes of different cellular organelles. DENV-2 appears to utilize only ER-derived structures [4] while WNV_KUN_ exploits the ER, ER-Golgi intermediate compartments, and *trans*-Golgi [20]. Consistent with DENV result, we observed co-localization of ER components with the viral dsRNA and NS1 protein, a component of the virus-IMS, to the WNV-IMS suggesting that WNV_NY99_ utilizes ER membrane as well. While our study and previously published data have demonstrated that components of the Golgi apparatus [20] localize to the WNV-IMS, we and others [38], [40] provide evidence to suggest that the biogenesis and recruitment of host proteins and/or existing membranes occur during the pre-Golgi step. It is possible that Golgi-resident proteins are sorted in the ER and get trapped in the WNV_KUN_-IMS as previously reported [38], [40]. This is supported by a recent publication showing that localization of the Golgi components to WNV_KUN_-IMS is coincidental rather than functional [40]. It is also probable that during IMS biogenesis the virus takes control of the secretory pathway to facilitate movement and maturation of virus particles along the secretory pathway [41] while the Golgi components get trapped in the ER. In the light of these data, it is not surprising that the WNV_NY99_ IMS biogenesis occurs at the ER exit-site. Interestingly, during infection of enterovirus, another positive strand RNA virus, virus-IMS form at the ER exit-site [28], suggesting this pattern of IMS localization appears probable to flavivirus.

### Intracellular localization of WNV_NY99_ NS4B in infected cells

The relationship of the flavivirus NS proteins (NS1, NS2A, NS3, NS4A and NS5) and the virus-IMS has been implicated solely based on immunostaining with distinct antibodies to different constellations of both virus-specific and host-specific proteins [16], [17], [20] but little is known regarding the relationship of NS4B protein and the virus-IMS. In this study we have demonstrated that during infection, WNV_NY99_ NS4B protein localizes to virus IMS defined by components of the flavivirus replication complexes including the dsRNA [4], [19], NS1 [16], [17], and envelope proteins [19]. Similar observations were made for DENV-2 [4], but not for WNV_KUN_ NS4B protein [14], [15], [16], [17], [42]. WNV_KUN_ NS4B was not detected in partly purified replicase complexes, except for NS1, NS2A, NS3, and NS4A [14]. The absence of WNV_KUN_ NS4B in virus IMS is not surprising since the major proportion of the protein translocates to the nucleus in infected and transfected cells [2], [3].

The significance of NS4B nuclear localization during the biosynthetic phase of WNV_KUN_ replication is only speculative. A computer-based analysis of the 255-amino acid sequence of WNV_NY99_ and WNV_KUN_ NS4B revealed seven amino acid differences between these strains. It is possible that the translocation of the WNV_KUN_ NS4B protein into the nucleus may be due to one or more of these amino acid differences. We have identified two amino acids at positions 29 (I to M) and 114 (S to A) (Fig. S2), which in concert changed the predicted WNV_KUN_ NS4B to assume exactly the same secondary structure as the WNV_NY99_ NS4B (data not shown). Our ongoing experiments are focused on this preliminary observation to study their role in NS4B nuclear localization.

### Alteration of the ER membrane in the generation of IMS by WNV_NY99_ NS4B in transfected cells

Although, previously published data have described mutations in NS4B leading to attenuation of the neuroinvasive and neurovirulence phenotypes in mice [10] or replication of the passage-adapted mosquito-borne flaviviruses [8], [9], [12], [43], the role of flavivirus NS4B in IMS generation is unknown. The localization of WNV_NY99_ NS4B to the ER-derived virus-IMS in our study suggests a role of this protein in IMS biogenesis. While the HCV NS4B is able to induce distinct membrane structures in transfected cells [44], DENV-2 NS4B and WNV_KUNV_ NS4B do not induce comparable structures after transfection of Huh-7 and Vero cells, respectively, with plasmid encoding the protein [2], [4], [6]. In agreement with HCV studies [4], we observed that WNV_NY99_ NS4B localizes to the ER. We have also demonstrated that the NS4B-IMS observed in the transfected cells is not a side effect of ER stress-induced apoptosis nor a general feature of overexpressed membrane protein further supporting that the NS4B-IMS we observed in the transfected cells represents characteristic structures identified by other investigators in flavivirus-infected cells [2], [13], [16], [20].

We demonstrated for the first time that NS4B induces membrane structures in transfected cells. Comparable membrane structures were formed when the NS4B plasmid was introduced into the infected cells suggesting that the NS4B-IMS represents at least one of many different membrane structures formed during virus infection. Even though comparable membrane structures were observed in the infected cells, previous studies showed that NS4B or NS4A could not be trans-complemented under the context of replication [45]. However, this claim remains elusive since deletion of region(s) within the viral polyprotein may have deleterious effects on polyprotein processing. Deleted regions within NS4A and NS4B of about 115 and 108 amino acids, respectively, from the WNV_KUN_ RNAs resulted in mutants that failed to be complemented when the corresponding proteins were provided in trans [45]. Interestingly, hepatitis C virus NS4B protein can trans-complement viral RNA replication and modulate production of infectious virus [46]. The failure to complement mutant WNV_KUN_ viral RNAs with the corresponding protein may be due to the effect of multiple viral proteins on RNA replication. Consistent with this suggestion several NS1 mutants in yellow fever infectious replicons were complemented when NS1 was supplied in trans but one mutant could not be complemented as the result of an eight C-terminal residues deletion of NS1 [47], shown to be required for cleavage at the NS1/2A site in DENV [48]. This C-terminal residues deletion of NS1 could perturb the function of NS2A or the downstream processing of the yellow fever polyprotein [47]. Moreover, the physical interaction between DENV NS4B and the helicase domain of NS3, a functional complex important for replication [32], was confirmed by biochemical pull-down and immunoprecipitation assays, both with purified proteins and with DENV-infected cell lysates, suggesting that transfected NS4B is functionally similar to wild type counterpart during infection [31].

Although it is possible to assume that transfected NS4A (or NS4B) functionally exchanges with the counterparts within the replication complex, the fact that NS4B or NS4A is recruited to the replication complex suggests that transfected NS4B or NS4A protein mimics its wild type counterparts. This argument is further validated by the fact that DENV NS4B purified from transfected cells or from infected cell lysates physically interacts with the helicase domain of NS3 [31], a functional complex important for replication [32]. Based on the above data, NS4A or NS4B could functionally be exchanged with the counterparts within the replication complex and possibly be trans complemented under the replication condition.

Previously published data have identified that a threshold level of RNA replication is required before membrane induction occurs [42] indicating that NS4B-IMS formation is dependent on achieving a critical intracellular concentration of the protein. While we demonstrated that the total NS4B protein concentration increased significantly over time, there was only a slight increase in NS4B-IMS forming cells. These data suggest that initiation of NS4B-IMS may require a minimum concentration threshold of NS4B protein but other mechanisms appear to be involved as well. This observed potential correlation of IMS formation with viral protein concentration threshold has been proposed by the published data on HCV NS4B [35].

### Role of the 2K-signal peptide during IMS formation

The 2K-signal peptide encompassing the COOH-terminal end of NS4A and preceding NS4B proteins has been highlighted in the literature to be important during establishment of flavivirus infection [22], from interferon antagonism [6], [49], cytokine and chemokine induction during DENV infection [7] to translocation of YFV NS4B into the ER lumen [21] and WNV_KUNV_ IMS formation [2]. Others have demonstrated that the 2K-signal peptide is not required for DENV-2 NS4B integration into the ER membrane [4] suggesting that the 2K may not be strictly required for membrane association and localization of NS4B protein to the ER in some of the flavivirus members. In agreement with this suggestion, we demonstrated that the WNV_NY99_ NS4B lacking the 2K-signal peptide is clearly associated with the ER membrane, is localized to the ER-derived virus IMS, and induces ER-derived membrane structures. These observations indicate that rather than significant involvement in the function of NS4B, the 2K may be important for the function of NS4A protein. Moreover, we did not observe an increase in NS4B-IMS forming cells when the 2K-signal peptide was retained indicating that NS4B contains internal sequences required for the initiation of the membrane structures. However, we cannot exclude the possibility that NS4B with and without 2K would form completely different topology on the ER membrane. Other investigators who attempted to determine NS4B topology by using DENV-infected cells were unsuccessful because the insertion of small epitope tags into different sites of NS4B led to the complete loss of viral RNA replication. The same studies also claimed that the unavailability of DENV NS4B-specific antibody directed against the potential cytoplasmic loop regions and other NS4B regions contributed to failure in determining NS4B topology using DENV-infected cells [4].

The computer-based prediction of the WNV NS4B protein using the SOSUI program [50] indicates that the number of transmembrane helices and topology of NS4B with or without 2K were same (Fig. 2 and S2). These data suggest that 2K does not influence the membrane association and topology of WNV NS4B. This is further supported by the biochemical and localization assays demonstrating that NS4B associates with the membrane (Fig. 6B), localizes to the ER (Fig. 6A) and induces membrane structures (Fig. 4A). These observations are consistent with previous reports [4], [21] suggesting that the functional significance of the 2K signal peptide in the topology of NS4B remains to be conclusively determined.

Previous studies suggest that WNV_KUN_ NS4A induces membrane structures resembling virus-IMS formed during infection [2], [19], [38] only when the 2K-signal peptide is retained, while removal of 2K results in the distribution of the NS4A protein to the Golgi apparatus [2]. In partial agreement with this observation, we demonstrated that WNV_NY99_ NS4A retaining 2K induced multiple membrane structures and is localized to the perinuclear region of the transfected cells. However, these membrane structures were not seen when the NS4A-2K plasmid was introduced into the infected cells. Rather, we observed diffused fluorescent patterns in the cytoplasm. It is possible that the viral protease, which cleaves the NS4A-2K junction, frees NS4A to accumulate undetected while the 2K-GFP protein disperses in the cytoplasm similar to control GFP cells. In agreement with our observation Miller *et al.* [19] have demonstrated that the NS4A lacking the 2K is the predominant species in the infected cells and individual expression of DENV NS4A lacking the 2K resulted in the induction of cytoplasmic membrane alterations resembling virus-induced structures. Therefore, it appears that NS4A of DENV and WNV_NY99_ is a component of the virus-IMS during infection but only when the 2K is cleaved by the viral protease.

### Regulation of IMS formation

There are three distinct characteristic membrane structures, including rough ER, CM/PC, and VP [13], during flavivirus infection. These structures are interconnected and provide a platform for viral RNA synthesis and virus assembly [38]. The mechanisms by which these different membrane structures consolidate to form the virus-IMS efficient for virus replication remain unknown. Others have demonstrated that the assembly of the nucleocapsid and immature virions occurs in the rough ER [38], [41], the VPs constitute the site of viral RNA synthesis [17], [51], and the CM/PC is the proposed site of protein translation and proteolytic processing of the viral polyprotein [17]. Each of these membrane structures contains a distinct set of NS proteins. The NS1, NS2A, NS3, NS4A, NS5, and dsRNA localize to the VP [16], [17], whereas NS2B, NS3, NS4A and NS5 localize to the CM/PC [16], [17]. It is important to note that NS3, NS4A, and NS5 localize to both VP and CM/PC structures but how these three proteins distribute between the VP and CM/PC structures is not entirely clear. Also, the viral protein responsible for VP formation is not clearly defined. While others have demonstrated that WNV_KUN_ NS4A induces membrane structures resembling CM/PC [2], [19], [38] and our studies suggest that WNV_NY99_ NS4B induces membrane structures resembling virus-IMS, it is plausible that NS4A can compensate for NS4B or *vice-versa* during virus infection. Our co-transfection studies indicate that NS4A and NS4B always remain co-localized in the IMS and associated with the same membrane fractions, which suggest that these proteins may function cooperatively in virus replication. Consistent with this suggestion Tajima *et al.* [52] have demonstrated that replication-defective DENV bearing mutations in NS4A is restored by additional mutations in NS4B. However, the nature of the interaction of these two proteins during IMS formation is unclear. The interaction may be a direct physical binding or dual participation in the same process, a phenomenon that has been demonstrated by others [53], which needs to be explored further. Other investigators have claimed that flavivirus NS3 is responsible for the formation of membrane structures that are not the sites for viral RNA synthesis [38], [39] but may be part of the entire IMS in virus-infected cells [2] which means that CM/PC is induced by NS3. Together, these observations suggest that specific viral proteins can initiate unique membrane structures that may consolidate, by some unknown mechanism, to form virus-IMS efficient for virus replication. Based on our demonstration of the co-localization of WNV_NY99_ NS4B with NS1, and with NS4A, further attests to the key role played by NS4B in the IMS formation. Since HCV NS4B is responsible for the aggregation of the vesicles, the building blocks for the VP and sites for viral RNA synthesis [54], we postulate that NS4B may be a critical regulatory protein during the consolidation of VP and CM/PC structures resulting in formation of fully functional virus-IMS. In support of this proposal Youn and colleagues [55] recently demonstrated a novel physical interaction between NS1, a protein that defines the VP structure [16], [17] and early RNA synthesis [47], and NS4B, a protein proposed in this study to play a key regulatory role in virus-IMS formation, and suggested a mechanism for how luminal NS1 conveys signal to regulate RNA replication, possibly from the VP structures to the cytoplasm where the CM/PC structures are located.

The reorganization of cellular membrane structures to site of WNV_NY99_ replication is complex but apparently NS1, NS3, NS4A, NS4B and possibly cellular factors contribute to various steps in this process. Future studies are warranted to define the exact role of NS4A- and NS4B-induced IMS in WNV replication and to determine whether any cellular proteins are recruited to the IMS. Information from such studies will be important for anti-WNV drug discovery.

## Materials and Methods

### Cells and virus

Low passage (6–11) HEK293 and Vero cells were cultivated in Dulbecco's modified Eagle medium supplemented with 10% fetal bovine serum (FBS) and M199 media supplemented with 5% FBS, respectively. WNV_NY99_ was employed for infection studies at a multiplicity of infection- (MOI) of 1 [56].

### Plasmid constructs

Standard molecular biology techniques were used for cloning [57]. All plasmids were confirmed by restriction digestion and sequence analysis using DNASTAR Lasergene 7.1 Sequence Analysis software (Madison, WI). RNA extracted from the WNV_NY99_ strain virus stock, which was originally isolated from crow brain and passaged once in Vero cells, was used as a template to generate the NS4A-2K-NS4B plasmid and various NS4A or NS4B fragments fused to the Cycle 3 green fluorescent protein (GFP) or harboring in TA cloning vectors pcDNA3.1/CT/NT-GFP-TOPO or pcDNA3.1/V5-His (Invitrogen). A PCR standard curve was generated using specific primers for WNV_NY99_ NS4B using thermal cycling conditions outlined in Table 1. All sense primers for C-terminal fusion constructs included an optimal translation initiation site and an ATG initiation codon. A GCT codon for alanine was located next to the ATG initiation codon preceding the DNA sequence to increase initiation efficiency (Table 1). Two NS4A expression plasmids, one lacking 2K (C-4A) and the other retaining 2K (C-4A-2K), were constructed into the pcDNA3.1/V5-His TOPO TA expression vector (Fig. 2). Similarly, several NS4B expression plasmids were constructed, the first retaining 2K (C-2K-4B) and the second lacking 2K (C-4B) fused at their COOH terminus to the cycle 3 GFP. Two additional plasmids were constructed, one directing the expression of the NS4A-2K-NS4B polyprotein (C-4AB), and the second containing 44-AA (C-sig4B), about 28% of NS4A (Fig. 2). Plasmid DNA was prepared from large-scale bacterial cultures and purified by cesium chloride (CsCl) equilibrium centrifugation [57]. MAX Efficiency Stbl2 *E. coli* strain (Invitrogen) was used for transformation. Nucleotide sequences of all constructs were confirmed at the Greenwood Molecular Biology Facility, University of Hawaii.

**Table 1:** Primer Sequences and Cycling Conditions.

Construct and Primer Name	Nucleotide Position*	Primer Sequence (5′-3′)	Tm °C	Amplicon Size	PCR Cycling Conditions
**C-4B** a **(1 to 255 aa)**					
4B-CT-F^b^	6916–6934	***acc acc atg g*** c ct AAC GAG ATG GGT TGG CTA G	68.3	777	95°C 5 min 1 cycle; 95°C 45 s, 64°C 45 s, 72°C 1 min, 30 cycles; 72°C 7 min 1 cycle
4B-CT-R^b^	7680–7658	G TCT TTT TAG TCC TGG TTT TTC CA	55.2		
**C-174B** a **(−17 to 255 aa)**					
17-4B-CT-F^b^	6865–6882	***acc acc atg g*** c ct CTA GCC GTG TTC CTG ATT	65.9	826	95°C 5 min 1 cycle; 95°C 45 s, 60°C 45 s, 72°C 1 min, 30 cycles; 72°C 7 min 1 cycle
**C-sig4B** a **(−44 to 255 aa)**					
sig-4B-CT-F^b^	6784–6801	***acc acc atg*** c GGA ATG TTG CTG CTC TCC	66.6	906	95°C 5 min 1 cycle; 95°C 45 s, 60°C 45 s, 72°C 1.5 min, 30 cycles; 72°C 7 min 1 cycle
**C-4AB** a **(−149 to 255 aa)**					
4AB-CT-F^b^	6469–6489	***acc acc atg g*** c ct TCT CAG ATA GGG CTC ATT GAG	65	1224	95°C 5 min 1 cycle; 95°C 45 s, 60°C 45 s, 72°C 2 min, 30 cycles; 72°C 7 min 1 cycle
**N-sig4B** a **(−44 to 255 aa)**					
si4B-NT-F^b^	6784–6801	GGA ATG TTG CTG CTC TCC C	58.1	900	95°C 5 min 1 cycle; 95°C 45 s, 55°C 45 s, 72°C 1 min, 30 cycles; 72°C 7 min 1 cycle
4B-NT-R^b^	7680–7658	TTC ATC TTT TTA GTC CTG GTT TTT CCA	53.5		

a Construct name. The numbers in parentheses indicate the NS4B amino acid (aa) from 1–255 and the minus sign represent the number of aa to C-teminal ends of NS4A preceding NS4B protein.

b Primer name. The CT or NT in the primer name denotes C- or N-terminal end of NS4B with respect to GFP fusion protein whereas the F or R denotes the sense or antisense primer, respectively.

c The Kozak sequence is in bold, lowercase and italicize. The nucleotides in lower case only are codon filling nucleotides. The viral sequences are in uppercase.

* GenBank Accession No. AF196835.

### Transient transfections and Expression

The reagent, PolyFect (Qiagen), was used to conduct transient transfections in 24-well plates, 6-well plates or coverslips with 1.0 µg plasmid DNA per 2.5×10^5^ cells, according to the manufacturer's protocol. The plasmid DNA was also transfected into the infected cells 1 hr after infection, as described above. This infection-transfection system was adopted from previously published data [28].

### Antibodies

Dilutions of primary and secondary antibodies used for immunostaining and western blotting are listed in Table 2. The anti- WNV_KUN_ and the anti-flavivirus FITC-conjugated antibodies were kindly provided by Dr. Edwin G. Westaway (Australia) and Dr. Duane J. Gubler (Singapore), respectively. The WNV anti-NS1 and -env mouse monoclonal antibodies were kindly provided by Dr. Michael S. Diamond (Saint Louis, MO).

**Table 2:** Antibodies used for imunostaining and Western blotting.

Protein	Primary Antibody	IS	WB	Secondary Antibody	IS	WB
Calnexin	rabbit anti-calnexin^a^	1∶200		Goat anti-rabbit IgG-TRITC^a^	1∶200	
			1∶2,000	Goat anti-rabbit IgG-AP^c^		1∶20,000
Protein Disulfide Isomerase (PDI)	rabbit anti-PDI^a^	1∶250		Goat anti-rabbit IgG-Alexa Fluor 594^b^	1∶20,000	
Glucose regulated protein 78 (grp78)	rabbit anti-grp78^a^		1∶1,000	Goat anti-rabbit IgG-AP^c^		1∶20,000
Green Fluorescent protein (GFP)	rabbit anti-GFP^b^		1∶5,000	Goat anti-rabbit IgG-AP^c^		1∶20,000
V5 epitope	mouse anti-V5^b^	1∶1,000		Goat anti-mouse IgG-Alexa Fluor 594^b^	1∶400	
			1∶5,000	Goat anti-mouse IgG-AP^c^		1∶20,000
double-stranded RNA (dsRNA)	mouse anti-J2^d^	1∶500		Goat anti-mouse IgG-Alexa Fluor 488^b^	1∶500	
WNV NS1	mouse anti-NS1	1∶200		Goat anti-mouse IgG-Alexa Fluor 555^b^	1∶400	
			1∶5,000	Goat anti-mouse IgG-AP^c^		1∶20,000
WNV Env	mouse anti-Env	1∶200		Goat anti-mouse IgG-Alexa Fluor 555^b^	1∶400	
			1∶5,000	Goat anti-mouse IgG-AP^c^		1∶20,000
Mannose-6-Phosphate Receptor (M6PR)	mouse anti-M6PR	1∶100		Goat anti-mouse IgG-Alexa Fluor 488^b^	1∶500	
Galactosyltransferase (GalT)	mouse anti-GalT	1∶100		Goat anti-mouse IgG-Alexa Fluor 488^b^	1∶500	
KDEL ER Marker	mouse anti-KDEL^f^	1∶100		Goat anti-mouse IgG-Alexa Fluor 488^b^	1∶500	
Golgi Marker 130 (GM130)	mouse anti-GM130^e^	1∶100		Goat anti-mouse IgG-Alexa Fluor 488^b^	1∶500	

a Sigma-Aldrich, Saint Louis, MO;

b Invitrogen Corporation, Carlsbad, CA;

c Bio-Rad Laboratories, Hercules, CA;

d English & Scientific Consulting, Hosok tere 2, H-3044 Szirak, Hungary;

e BD Transduction Laboratories, BD Biosciences, San Jose, CA;

f Abcam, Cambridge, MA.

IS, Immunostaining; WB, Western blotting; AP, Alkaline Phosphatase; TRITC, Tetramethyl rhodamine isothiocyanate.

### Indirect immunofluorescence test

For detection of PDI, cells were fixed with 4% paraformaldehyde in PBS and permeabilized in 0.5% Triton X-100. For detection of calnexin, cells were fixed with pre-cooled methanol and permeabilized with pre-cooled acetone, washed and incubated with the appropriate primary and secondary antibodies (Table 2), as described previously [3]. IF detection of the Golgi apparatus, early endosome and WNV_NY99_ NS4B were conducted as described previously [3], [28], [58], [59]. Slides were viewed and captured using Olympus confocal microscope. The images were processed (image, adjustments, and levels) with the Adobe Photoshop CS3 Version 10.0.1 according to the policy formulated by the Digital Image Processing & Ethics Group of the Microscopy Society of America (MSA) Education Committee and was adopted as MSA policy at the Summer Council meeting August 2–3, 2003.

### Quantitation of IMS-forming cells

Cells transfected with various plasmids were fixed with 4% PFA on coverslips and viewed using a Zeiss inverted microscope. The number of IMS-forming cells were recorded and converted into percentage of total number of GFP-positive cells per field (100 to 300 GFP-positive cells per field and ten to fifteen microscopic fields per treatment) (Fig. 5A, top panel). The efficiency of transfection were also calculated and converted into percentage of total number of cells per field (Fig. 5A, bottom panel). Transfected cells were fixed as described above and labeled with DAPI viewed with the 20× objective and the IMS-forming cells were confirmed with 63× objective.

### Cell lysis

The cell pellet was resuspended in the appropriate lysing buffer and phase separation of the soluble proteins from membrane proteins was conducted as described previously [60]. Briefly, cells were lysed on ice with 1% Triton X-114 and 1% proteinase inhibitor cocktail (Sigma-Aldrich). Proteins were separated by layering the lysate on 6% sucrose in a buffer solution at 30°C. The aqueous and detergent phases, as well as input lysate (before separation), were analyzed by WB assay.

### Subcellular fractionation

HEK293 cells co-transfected with C-4A and C-4B, were fractionated as described previously [36], [37] with modification. Briefly, cells of two confluent 100-mm plates were combined by scraping into 400 µL of ice-cold hypotonic buffer containing 1% proteinase inhibitor cocktail (Sigma-Aldrich). The cells were homogenized by 20 passages through a 25-gauge needle, and the post-nuclear supernatant (PNS) was prepared by centrifugation for 10 min at 600 g. Aliquots of ≈400 µL of PNS were layered on a discontinuous sucrose density gradient (0.2, 0.4, 0.6, 1.0, 1.4, and 1.8 M, 470 µL each) and centrifuged for 2 h at 55,083 g Beckman SW55 rotor). Eleven fractions, ≈292 µL each, were collected from the top. Twenty-five µL each fraction was separated by SDS/PAGE.

### Western blotting assay

Cell lysates were electrophoresed on 4–12% NuPAGE gels (Invitrogen) transferred onto a nitrocellulose membrane and incubated with primary antibodies (Table 2) [61] followed by incubation with either alkaline phosphatase (AP)-conjugated or horseradish peroxidase (HRP)-conjugated secondary antibody. The enzyme activity was detected as described previously.

### Statistical analyses

The Kruskel Wallis test, a non-parametric version of ANOVA, was employed to determine differences between treatments, and the non-parametric Mann Whitney test was also employed to compare individual differences.

## Supporting Information

Figure S1 **Fluorescence patterns and expression of two ER membrane associated proteins.** HEK293 cells were transfected with a (A) SelN-GFP or (B) KDEL-RFP plasmid and fixed or harvested at 12, 24 and 40 hr after transfection. Fifty µg of harvested cell lysates were electrophoresed on SDS-PAGE followed by immunoblotting with rabbit anti-GFP or mouse anti-KDEL monoclonal antibody followed by a peroxidase-conjugated species-specific secondary antibody. The western blot image was analyzed using ImageJ to determine the relative intensity of (A) SelN or (B) KDEL expression level at 12, 24 and 40 hr after transfection relative to β-actin, shown as a percent relative intensity. Scale bar, 10 µm.
(TIF)

Figure S2 **Predicted secondary structure of WNV NS4B.** In-silico aa substitutions in WNV_KUNV_ NS4B mimics WNV_NY99_ NS4B. WNV NS4B transmembrane helical segments (THS) were predicted by SOSUI secondary structures tool. The aa positions of the THS in WNV NS4B protein are indicated. The two horizontal lines represent the ER double membrane while the freeform lines indicate the NS4B segments outside the membranes. The rectangular boxes depict the THS and the numbers indicate the beginning and end of each THS segment. Amino acid substitutions of WNV_KUNV_ NS4B were made at positions 29 (isoleucine-methionine) and 114 (serine-alanine) as indicated by the arrows. C, Cysteine; I, isoleucine; M, methionine; S, serine; A, alanine.
(TIF)
