# Supplementary figures and images for: Induction of Endoplasmic Reticulum-Derived Replication-Competent Membrane Structures by West Nile Virus Non-Structural Protein 4B
Source: PLoS One. 2014 Jan 20;9(1):e84040. doi: 10.1371/journal.pone.0084040 (PMC3896337; doi:10.1371/journal.pone.0084040)

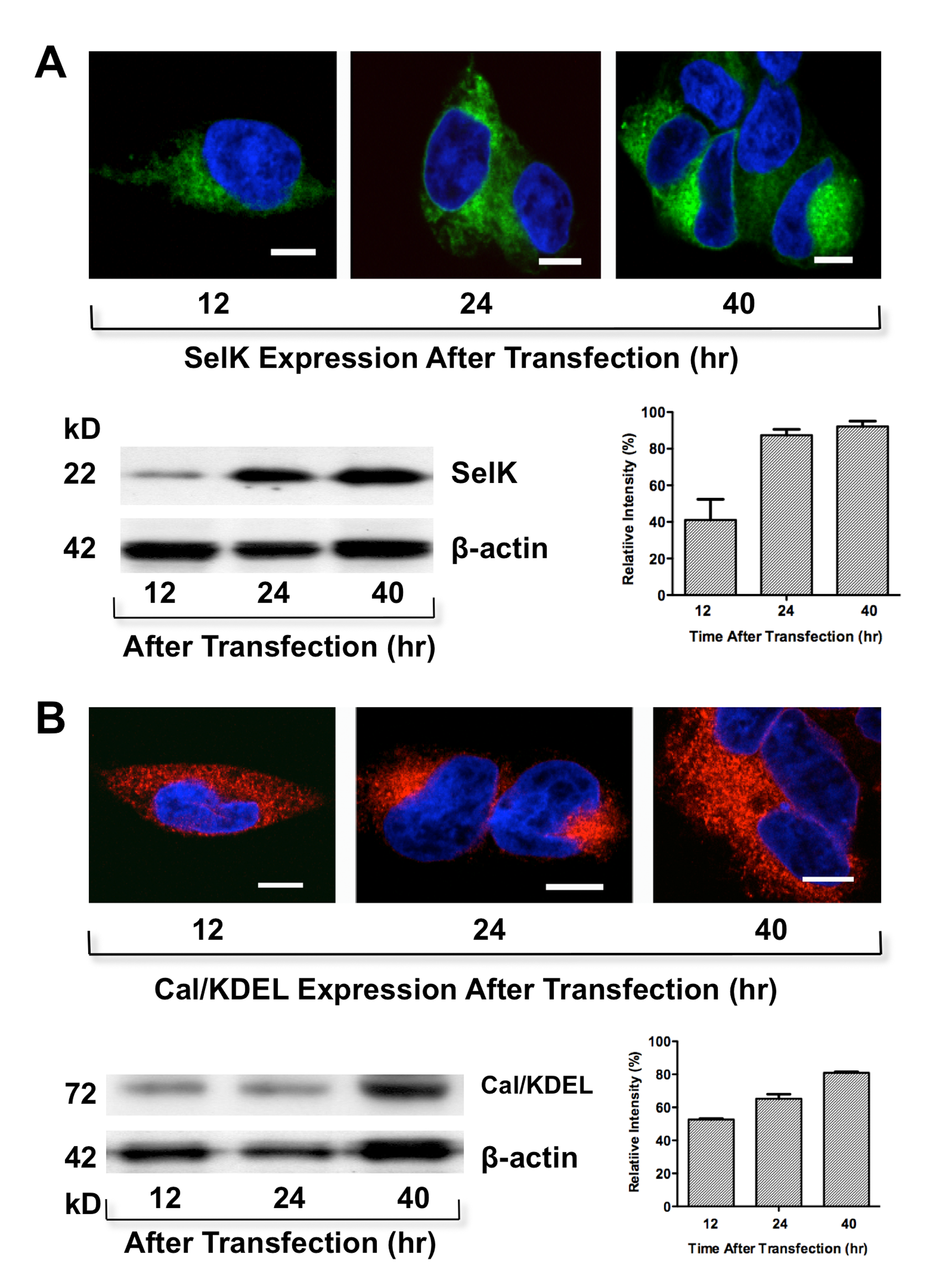

Supplement: Figure S1 — Fluorescence patterns and expression of two ER membrane associated proteins. HEK293 cells were transfected with a (A) SelN-GFP or (B) KDEL-RFP plasmid and fixed or harvested at 12, 24 and 40 hr after transfection. Fifty µg of harvested cell lysates were electrophoresed on SDS-PAGE followed by immunoblotting with rabbit anti-GFP or mouse anti-KDEL monoclonal antibody followed by a peroxidase-conjugated species-specific secondary antibody. The western blot image was analyzed using ImageJ to determine the relative intensity of (A) SelN or (B) KDEL expression level at 12, 24 and 40 hr after transfection relative to β-actin, shown as a percent relative intensity. Scale bar, 10 µm. (TIF) [file pone.0084040.s001.tif]

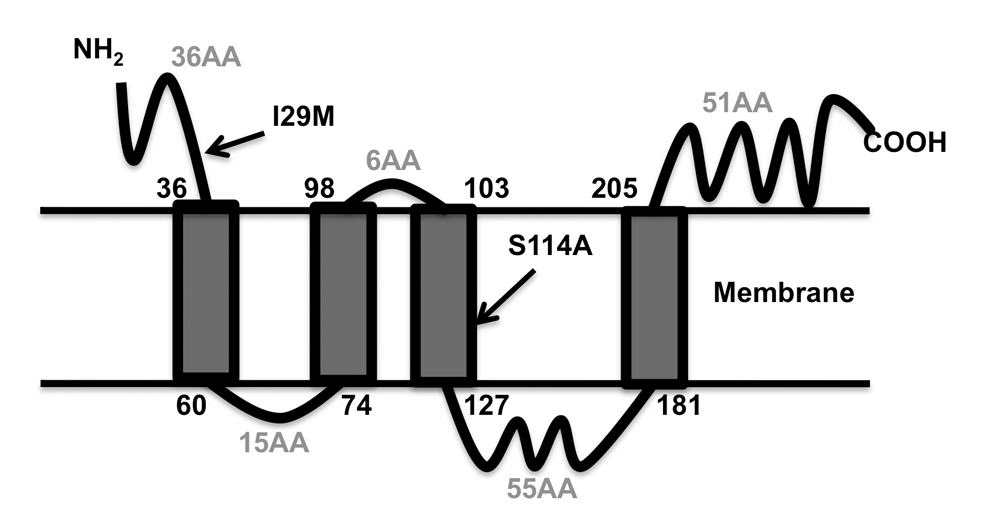

Supplement: Figure S2 — Predicted secondary structure of WNV NS4B. In-silico aa substitutions in WNVKUNV NS4B mimics WNVNY99 NS4B. WNV NS4B transmembrane helical segments (THS) were predicted by SOSUI secondary structures tool. The aa positions of the THS in WNV NS4B protein are indicated. The two horizontal lines represent the ER double membrane while the freeform lines indicate the NS4B segments outside the membranes. The rectangular boxes depict the THS and the numbers indicate the beginning and end of each THS segment. Amino acid substitutions of WNVKUNV NS4B were made at positions 29 (isoleucine-methionine) and 114 (serine-alanine) as indicated by the arrows. C, Cysteine; I, isoleucine; M, methionine; S, serine; A, alanine. (TIF) [file pone.0084040.s002.tif]
